# Epidemiology and treatment of Behçet’s disease in the USA: insights from the Rheumatology Informatics System for Effectiveness (RISE) Registry with a comparison with other published cohorts from endemic regions

**DOI:** 10.1186/s13075-021-02615-7

**Published:** 2021-08-30

**Authors:** Nevin Hammam, Jing Li, Michael Evans, Julia L. Kay, Zara Izadi, Christine Anastasiou, Milena A. Gianfrancesco, Jinoos Yazdany, Gabriela Schmajuk

**Affiliations:** 1 Division of Rheumatology, Department of Medicine, University of California San Francisco, San Francisco, CA USA; 2 Philip R. Lee Institute for Health Policy Research, San Francisco, USA; 3 San Francisco Veterans Affairs Medical Center, 4150 Clement St 111R, San Francisco, CA 94121 USA

**Keywords:** Behçet’s disease, RISE, ECR, Endemic regions, Medication

## Abstract

**Background:**

Behçet’s disease (BD), a chronic systemic vasculitis, has distinct geographical and ethnic variation. Data regarding the epidemiology of patients with BD in the U.S. are limited; therefore, we sought to describe BD patient characteristics and medication use in the U.S., and compared them with data from patients from endemic regions.

**Methods:**

We conducted a cross-sectional study using data from the RISE registry (2014–2018). Patients aged ≥ 18 years with BD were included. Sociodemographic and treatment information was extracted. We compared patients from the RISE registry to data from other published studies of patients with BD from endemic areas.

**Results:**

One thousand three hundred twenty-three subjects with BD from the RISE registry were included. Mean age was 48.7 ± 16.3 years, female to male ratio was 3.8:1, and 66.7% were White. The most frequently used medications included glucocorticoids (67.6%) and colchicine (55.0%). Infliximab and adalimumab were the most used biologics (14.5% and 14.1%, respectively); 3.2% of patients used apremilast. The RISE registry had more women (79.3%), and patients were older compared to previously published BD studies from endemic areas. Methotrexate and TNFi were more commonly reported in RISE (21.8% and 29.4%) compared to studies from Egypt and Turkey. Colchicine, cyclosporine, and cyclophosphamide were more commonly used in cohorts from Egypt, Turkey, and Iran.

**Conclusions:**

Findings from the largest BD dataset in the U.S. suggest that BD patients are predominantly female. Further research is needed to explore the reasons for the higher prevalence of BD among women in the U.S. and its possible impact on disease severity and management.

**Supplementary Information:**

The online version contains supplementary material available at 10.1186/s13075-021-02615-7.

## Background

Behçet’s disease (BD) is a chronic multisystem vasculitis that can increase morbidity and mortality. The prevalence of the disease, the frequency of specific clinical findings, and the mortality rate have distinct geographical and ethnic variation: BD prevalence is higher in the Middle East and East Asia but remains rare in North America [1]. In the United States (U.S.), the estimated prevalence ranges from 0.33 to 5.2 people per 100,000 population [2]. A male predominance with a severe disease course has been observed in Arab populations [3], while female predominance has been reported in 2 small studies of patients in the U.S. [4, 5].

The prevalence of BD in the U.S. is increasing, which may be due to increased disease recognition and immigration from endemic areas [2]; however, robust epidemiologic data about BD in the U.S. is scarce. While descriptive cohort studies of BD in the U.S. exist, they are from single centers, limited by small sample sizes, and have not reported data from multiple racial or ethnic groups [2, 4–6]. Furthermore, previous studies comparing the characteristics of patients with BD from the northeastern U.S. with patients from Turkey [5] and Iran [7] raised the possibility that BD in U.S. patients may have unique features compared to typical BD populations. However, to date, no national studies of BD in the U.S. have been published.

To better investigate the demographic characteristics, comorbidities, and medical treatment patterns of BD in the U.S., we used data from the Rheumatology Informatics System for Effectiveness (RISE). RISE is a national electronic health record (EHR) registry that captures all patients seen by participating U.S. rheumatologists [8]. We also compared the available RISE-BD data with published epidemiological studies of BD from endemic regions.

## Materials and methods

### Data source, timelines, and study population

This observational study was conducted using data from the RISE Registry (2014–2018). RISE, a national electronic health record (EHR)-enabled rheumatology registry, collects data on all patients seen during routine outpatient clinical care in participating rheumatology practices across the U.S., reducing the selection bias present in single insurer-based studies [8]. As of 2018, RISE held validated data from 1113 providers in 226 practices, representing more than 30% of the U.S. clinical rheumatology workforce. Available data is collected through the EHR and includes individual demographics, diagnoses, procedures, medications, laboratory test results, and vital signs. Patients in RISE aged 18 years and older with at least 2 diagnosis codes (International Classification of Diseases, Ninth Revision, Clinical Modification (ICD-9-CM) 136.1 or ICD-10-CM M35.2) for BD at least ≥ 30 days apart at any time during the study period were included [9].

### Covariates

We extracted information on patient characteristics from the RISE registry. Patient characteristics included age, gender, race/ethnicity (White, Hispanic/Latino, Black/African American/other), geographic region of residence (East North Central, West North Central, Mid-Atlantic, Mountain, New England, Pacific, South Atlantic, East South Central, and West South Central), insurance type (private, Medicare, any Medicaid, other) when available, and the number of rheumatology visits during the study period. We examined clinical comorbid conditions, including a diagnosis of diabetes, asthma, hypertension, and osteoporosis. We also calculated the Deyo-Charlson Comorbidity Index (CCI) score using data recorded at any time during the study period [10].

### Medications

Medications potentially related to BD were identified by either Generic Product Identifier codes or National Drug Codes. The following categories were identified (1) conventional synthetic DMARDs (csDMARDs): methotrexate, azathioprine, hydroxychloroquine, leflunomide, sulfasalazine, mycophenolate mofetil, cyclophosphamide, cyclosporine, minocycline, and tacrolimus; (2) tumor necrosis factor (TNF) inhibitors: etanercept, infliximab, adalimumab, golimumab, and certolizumab; (3) non-TNF biologics: abatacept, rituximab, secukinumab, ustekinumab, omalizumab, anakinra, canakinumab, tocilizumab, and sarilumab; (4) targeted synthetic DMARDs (tsDMARDs): tofacitinib, baricitinib, and apremilast; (5) systemic glucocorticoids including prednisone and other oral and intravenous steroids; and (6) anticoagulants including warfarin, rivaroxaban, apixaban, enoxaparin, dabigatran, and edoxaban.

### BD studies from endemic regions

We also reviewed the published studies to identify reports of BD from endemic regions. We searched PubMed for studies of “Behçet’s disease” from 2010 to present (2020). Studies were eligible if they (1) reported on adults with BD; (2) were published in English; (3) included countries along the ancient Silk Road, extending from Japan to the Middle East, or Mediterranean countries including Turkey and Iran; (4) reported on ≥ 100 BD patients; and (5) had information on demographic factors and medication use. In studies that included only specific BD phenotypes (uveitis, or neuropsychiatric BD) or that included patients younger than 18 years, we excluded these studies. For each included study, we abstracted the following data: number of patients, country, patients’ age and gender, and related medication use. We did not abstract any clinical manifestations because of insufficient clinical data in the RISE-BD records.

### Statistical analysis

Descriptive statistics including mean and standard deviation (SD), and median and interquartile range (IQR), were reported for continuous variables, while frequency and percent were reported for categorical variables to describe BD patients within the RISE registry and those included in studies from endemic regions. Comparisons of the proportion of patients using different medications between men and women in the RISE registry were performed using chi-square tests. A *p*-value of less than 0.05 was considered statistically significant. All data analyses were conducted using SAS 9.4 (SAS institute, Cary, NC), and Stata statistical software version 15 (StataCorp). For privacy protections, we reported no cell sizes < 10. The Western IRB and UCSF Committee on Human Research approved this study.

## Results

### Characteristics of BD patients in the U.S.

A total of 1323 subjects with BD from the RISE registry were analyzed. The female to male ratio was 3.8:1, with a mean age of 48.7 (SD = 16.3) years. The majority of patients were white (66.7%) which is similar to the underlying population of all RISE patients (64.9%). The median duration of follow-up in the registry was 2.5 (IQR 0.9, 4.4) years (Table 1). Most patients were enrolled in a private health plan (42.1%) or Medicare (15.5%). Osteoporosis (11.7%), hypertension (8.8%), and dyslipidemia (4.7%) were among the most commonly observed comorbid conditions. The mean Deyo-Charlson Index score was 0.55 (SD = 1.0); 9.3% of patient had a score equal to or more than 2.

**Table 1:** Characteristics of patients with Behçet’s disease in the RISE registry

Characteristics		Total patients (*N* = 1323)
Age, mean (SD)		48.7 (16.3)
Sex (female), *N* (%)		1049 (79.3)
Race, *N* (%)	White	882 (66.7)
	Hispanic or Latino	72 (5.4)
	Black or African American	65 (4.9)
	Asian	34 (2.6)
	Other^a^	106 (11.9)
	Missing	164 (12.4)
Insurance, *N* (%)	Medicare	205 (15.5)
	Private	557 (42.1)
	Medicaid	43 (3.2)
	Other^b^	75 (5.7)
	Missing	443 (33.5)
U.S. geographic division, *N* (%)	East North Central	22 (1.7)
	West North Central	153 (11.6)
	Mid-Atlantic	159 (12.0)
	Mountain	109 (8.2)
	New England	396 (29.9)
	Pacific	116 (8.8)
	South Atlantic	153 (11.6)
	East South Central	62 (4.7)
	West South Central	144 (10.9)
	Missing	< 10
Comorbidities, *N* (%)	Osteoporosis	155 (11.7)
	Hypertension	117 (8.8)
	Hyperlipidemia	62 (4.7)
	Cerebrovascular accidents /TIA	51 (3.7)
	Diabetes mellitus	48 (3.6)
	Charlson comorbidity index score, mean (SD)	0.55 (1.0)
	Charlson comorbidity index score	
	1	339 (25.6)
	≥ 2	123 (9.3)
Clinical characteristics	Number of visits in RISE, median (IQR)	4.5 (2.5, 8)
	Duration of follow-up time (years), median (IQR)	2.5 (0.9, 4.4)

*RISE* Rheumatology Informatics System for Effectiveness, *TIA* transient ischemic attack, *IQR* interquartile range

^a^Other race: Not determined OMB race, and American Indian or Alaska, Native Hawaiian

^b^Other insurance: include Veteran

The distribution of age, sex, and ethnicity of patients with BD was different compared to the underlying population of patients in the RISE registry (Supplemental Table 1). The *F* to *M* ratio was higher in BD patients compared to the overall RISE population (3.8:1 vs 2.8:1, *p* < 0.0001). We found a significantly higher proportion of Asian patients with BD compared to the overall RISE population (2.6% vs 1.6%, *p* < 0.0001). In contrast, there was a lower proportion of Black patients (4.9 vs. 7.2, *p* < 0.0001).

**Fig. 1:**
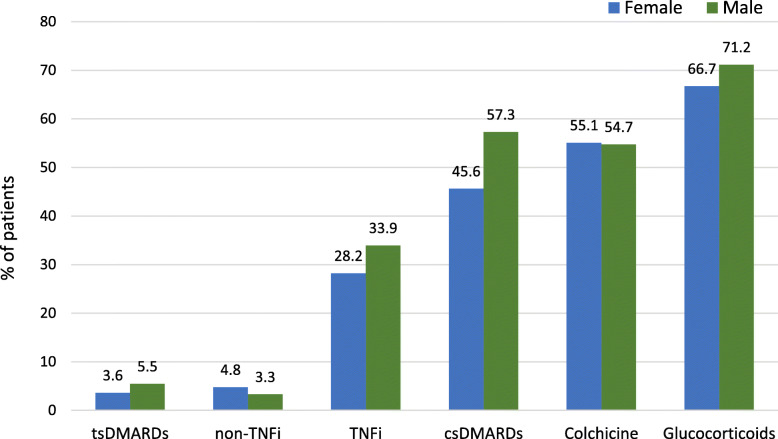
The frequencies of medication use among patients with BD from the RISE registry, stratified by sex. There were no significant differences in the proportion of patients using each class of medication between men and women

The systemic medications used to manage BD in the RISE registry are shown in Table 2. The most frequently used medications included glucocorticoids (67.6%) and colchicine (55.0%). Infliximab and adalimumab were the most commonly used biologics (14.5% and 14.1%, respectively); 3.2% of patients used apremilast. There were no significant differences in the proportion of patients using each class of medication between men and women, Fig. 1.

**Table 2:** Medications administered to patients with Behçet’s disease in the RISE registry

Medications		Total patients (*N* = 1323)
No therapy recorded		111 (8.4)
Colchicine		728 (55.0)
Dapsone		62 (4.7)
Glucocorticoids	Any prednisone or equivalent^a^	895 (67.6)
csDMARDs	Azathioprine	418 (31.6)
	Methotrexate	288 (21.8)
	Hydroxychloroquine	117 (8.8)
	Sulfasalazine	53 (4.0)
	Mycophenolate	31 (2.3)
	Leflunomide	26 (2.0)
	Cyclosporine	23 (1.7)
	Tacrolimus	11 (0.83)
	Cyclophosphamide	< 10
	Minocycline	< 10
Biologics-TNFi	Infliximab	192 (14.5)
	Adalimumab	187 (14.1)
	Etanercept	92 (6.9)
	Certolizumab	30 (2.3)
	Golimumab	20 (1.5)
Biologics-non-TNFi^b^		59 (4.5)
Targeted small molecules	Tofacitinib	11 (0.80)
	Baricitinib	< 10
	Apremilast	42 (3.2)
Anticoagulants	Warfarin	39 (2.9)
	Rivaroxaban	27 (2.0)
	Apixaban	15 (1.1)
	Enoxaparin	11 (0.8)
	Dabigatran	< 10
	Edoxaban	0 (0.0)

*RISE* Rheumatology Informatics System for Effectiveness, *csDMARDs* conventional disease modifying anti-rheumatic drugs, *TNFi* tumor necrosis factor inhibitors

For privacy protection, we reported no cell sizes < 10

^a^Prednisone or equivalent included prednisone and other oral and intravenous steroids

^b^Biologics-non-TNFi include rituximab, abatacept, tocilizumab, ustekinumab, anakinra, and secukinumab

We found that 78 (5.9%) of BD patients were using at least one of the anticoagulants. The most frequently used anticoagulant was warfarin followed by Rivaroxaban in 3.0% and 2.0% of BD patients, respectively.

### Comparison of BD patients between the U.S. and studies from endemic areas

Sociodemographic and medication information extracted from four published studies that met the inclusion criteria described above: Egypt (Egyptian College of Rheumatology (ECR) Registry) (*N* = 1526), Turkey (*N* = 682), Iran (*N* = 163), and Japan (*N* = 135) [7, 11–13]. Data from the U.S. and BD cohorts from endemic regions is shown in Table 3. In general, the RISE registry had more women with BD compared to all studies from endemic regions (79.3% in RISE vs. 27.8% in the Egyptian cohort, and 16.6% in the Turkish cohort, 38.7% in the Iranian cohort, and 57.8% in the Japanese cohort) and patients were older (mean age (SD); 48.7 (16.3) in RISE vs. 35.7 (9.84) in ECR and 33.9 (9.9) in Turkish). There were differences in medication use, with methotrexate and TNFi used more commonly used in RISE patients (21.8% and 29.4%) compared to the ECR (7.2% and 8.3%). Colchicine and cyclosporine were more commonly used in other cohorts compared to RISE patients. Fewer patients in RISE were treated with glucocorticoids than in Egypt (67.6% vs 90.2%), although we did not observe these differences in the Iranian cohort (67.5%).

**Table 3:** Summary of the sociodemographic characteristics and treatments administered to patients with Behçet’s disease in the RISE (U.S.) registry and studies from endemic regions

Characteristics		Patients with BD in U.S. (RISE) (*N* = 1323)	Patients with BD in Egypt (ECR) [11] (*N* = 1526)	Patients with BD in Turkey [12] (*N* = 682)	Patients with BD in Iran [7] (*N* = 163)	Patients with BD in Japan [13] (*N* = 135)
**Sociodemographic**	Age, mean (SD)	48.7 (16.3)	35.7 (9.84)	33.0 (9.9)	NR	NR
	Female, *N* (%)	1049 (79.3)	424 (27.8)	113 (16.6)	63 (38.7)	78 (57.8)
**Medications**	Colchicine, *N* (%)	728 (55.0)	611 (82.7)	599 (87.8)	108 (66.3)	90 (66.7)
	Dapsone, *N* (%)	62 (4.7)	NR	NR	NR	0 (0.0)
	Glucocorticoids, *N* (%)	895 (67.6)	947 (90.2)	384 (56.3)	110 (67.5)	78 (57.8)
	**cDMARDs,*N*(%)**					1 (0.70)
	Azathioprine, *N* (%)	418 (31.6)	474 (26.7)	347 (50.8)	48 (29.4)	17 (12.6)
	Methotrexate, *N* (%)	288 (21.8)	67 (7.2)	14 (2.1)	51 (31.3)	9 (6.7)
	Hydroxychloroquine, *N* (%)	117 (8.8)	NR	NR	NR	NR
	Sulfasalazine, *N* (%)	53 (4.0)	NR	51 (7.5)	3 (5.4)	31 (23.0)
	Mycophenolate, *N* (%)	31 (2.3)	NR	NR	0 (0.0)	0 (0.0)
	Leflunomide	26 (1.9)	NR	NR	NR	NR
	Cyclosporine, *N* (%)	23 (1.5)	282 (26.7)	93 (13.6)	13 (8.0)	14 (10.4)
	Cyclophosphamide, *N* (%)	< 10	208 (20.1)	39 (5.7)	39 (23.9)	0 (0.0)
	Minocycline, *N* (%)	< 10	NR	NR	NR	NR
	Tacrolimus, *N* (%)	11 (0.83)	NR	NR	NR	NR
	**Biologics-TNFi,*N*(%)**	389 (29.4)	83 (8.3)	NR	1 (0.60)	NR
	Infliximab, *N* (%)	192 (14.5)	NR	4 (0.6)	NR	NR
	Adalimumab, *N* (%)	187 (14.1)	NR	NR	NR	10 (7.4)
	Etanercept, *N* (%)	92 (6.9)	NR	NR	NR	1 (0.70)
	Golimumab, *N* (%)	20 (1.5)	NR	NR	NR	0 (0.0)
	Certolizumab, *N* (%)	30 (2.3)	NR	NR	NR	NR
	**Biologics-non-TNFi**^**a**^**,*N*(%)**	59 (4.5)	NR	NR	NR	NR
	**tsDMARDs**	53 (4.0)	NR	NR	NR	NR

*RISE* Rheumatology Informatics System for Effectiveness, *ECR* Egyptian College of Rheumatology, *csDMARDs* conventional disease modifying anti-rheumatic drugs, *TNFi* tumor necrosis factor inhibitors, *NR* non recorded

^a^Biologics-non-TNFi include rituximab, abatacept, tocilizumab, ustekinumab, and secukinumab

## Discussion

Although Behçet’s disease is rare in North America, this report from the RISE registry includes the largest dataset of U.S. patients with BD to date. Among 1323 BD patients seen by U.S. rheumatologists, BD patients were more likely to be female, thus confirming previous reports from smaller studies [2, 4–6]. Nearly one third of BD patients in the RISE registry used TNFi, which is higher than has been previously reported and significantly higher than reported in studies from endemic regions. Consistent with prior reports, BD has a higher prevalence among Asian patients, and it has rarely been reported in the Black population [1, 14]. One potential explanation is the high prevalence of HLA-B*51 among Asians, an allele that has also been implicated in BD [14].

The female predilection (79.3%) in the current study is consistent with previous reports of U.S.-based studies (64–80%) [5, 7, 13, 15]. The sex distribution found in RISE data is in contrast to reports from Eastern and Middle East countries that demonstrate a male predominance [16]. The reasons underlying sex distribution differences remain largely unknown but are likely multifactorial. This may be attributed in part to a possible cultural reluctance among women from Eastern countries to visit a physician for genital ulcers leading to underestimation of women with BD, which is supported by the lower prevalence of genital ulcers reported in these countries [17]. However, it is unclear whether the observed difference in sex predilection represents women using more healthcare in the U.S. [18] compared to other countries or a true difference in the epidemiology of BD due to environmental or geographic factors.

Prior studies have also shown differences in the prevalence of specific BD clinical manifestations between the sexes [19, 20]. We attempted to extract information about clinical manifestations of BD from RISE data, but we found that they were severely under coded in the EHR by rheumatologists, including a low number of ICD codes for oral and genital ulcers and chronic uveitis even among patients with multiple codes for BD (data not shown). Other studies have also noted underestimation of specific clinical phenotypes using EHR data [21], including in other rheumatic conditions [22]. Prior BD studies identified manifestations using medical record review [2, 13]; however, within the RISE registry, it is not possible to perform systematic chart reviews for patients at this time. In the future, a combination of text mining and manual review of clinical text to extract information may allow for more detailed identification of specific disease manifestations in the RISE registry [23, 24]. In addition, the fact that BD patients may seek care from clinicians across many different specialties, including neurology, dermatology, ophthalmology, and rheumatology makes it challenging to gain a complete view regarding clinical manifestations from single specialty EHR records. Research into different presentation of BD in nonendemic areas may provide new clues to the pathogenesis of this condition.

Given the heterogeneity of BD, treatment approaches are highly variable and based on the severity of organ involvement and patient preferences [25]. An obstacle for treating patients with BD is the lack of the Food and Drug Administration (FDA) approved therapies; aside from apremilast (July 19, 2019), all other therapeutic options are prescribed off-label. The systemic medications used to manage Behçet’s disease largely consist of glucocorticoids and colchicine. In the RISE registry, a large proportion of patients (67.6%) were treated with systemic glucocorticoids, as noted in other U.S. cohorts (34.0–82.1%) [5, 7, 15]. Similar to previous reports, about one third of patients were treated with newer therapies including biologics such as TNFi drugs [5, 7, 15]. Also, non-TNFi drugs such secukinumab and ustekinumab were used by some rheumatologists; data on these agents are limited [26, 27]. The present study is also the first real-world setting reporting the use of apremilast in the treatment of BD patients (3.2%), even the study period was prior to the FDA approval of apremilast for BD mouth ulcers. To date, apremilast, a phosphodiesterase 4 (PDE4) inhibitor, is the only drug currently approved by the U.S. FDA for the treatment of oral ulcers associated with BD [28].

The difference in use of biologic medications between the U.S., and published data from endemic regions may be driven by many factors. First U.S. patients seen by rheumatologists may have more joint complaints, as has been described in other studies [7, 13], and biologics would be well-suited for these symptoms. Second, insurance systems are likely different between the U.S. and Eastern countries, which may make biologic medications more accessible in the U.S. Rheumatologists preferred the choice of anti-TNFi therapy over conventional DMARDs for BD when cost and prior authorization issue were not a concern [29]. Third, U.S. primary care physicians may not be as familiar with this rare disease; thus, only the most complicated patients may be diagnosed and treated by rheumatologists. The magnitude of the regional impact on treatment differences remains difficult to assess, mainly because we were unable to explore the clinical manifestation of BD in this study.

There are several strengths of this study. We report on the largest collection of adult BD patients in the U.S. using a nationwide registry. These data provide real-world evidence of disease management rather than being limited to single, academic centers. However, our study has several potential limitations. First, diagnosis codes used in the identification of BD are good indicators but do not guarantee the presence of disease, although Lenert et al. [9] examined the validity of ICD codes to identify BD patients and found that the positive predictive value was excellent (> 99%). Second, as discussed above, we did not have access to information on BD clinical manifestations. Third, one potential limitation of the data related to anticoagulants is the multiple possible indications for their use. We cannot be certain of the indication of anticoagulants used among included patients as these drugs are commonly prescribed in the management of non-thrombotic conditions, e.g., primary prevention of stroke in atrial fibrillation. Fourth, a direct comparison of RISE-BD vs non-RISE studies was complicated by differences in the methods of studies performed and the availability of data collected. Other limitations in the comparison could be due to differences in sample sizes. Future work should explore whether differences in clinical manifestations can explain the differences in treatment strategies.

## Conclusion

In conclusion, the RISE registry captured data from 1323 patients with BD distributed across all races/ethnicities and geographic regions of the U.S. The current study represents the largest dataset of U.S. BD patients reported to date. We confirmed the female predominance in the U.S. and found that a substantial proportion of BD patients were taking a biologic medication. In comparison to patients in endemic regions, U.S. BD patients were older and received more biological medications, which raises the possibility that there are a variety of epidemiologic and clinical differences in disease between patients in the U.S. and those from endemic areas. Future studies examining clinical manifestations among large numbers of U.S. BD patients are needed.

## Supplementary Information

**Additional file 1:.** Supplementary Table 1. Characteristics of patients with BD compared with all patients in the RISE registry.

## Data Availability

The American College of Rheumatology (ACR) owns the data in the RISE registry, and UCSF, as a Data Analytic Center for the ACR, has access to the data for specific research projects, including this one, but is contractually obligated to not share this data, even in a de-identified state.

## Abbreviations

BD: Behçet’s disease
CCI: Charlson Comorbidity Index
csDMARDs: Conventional synthetic DMARDs
ECR: Egyptian College of Rheumatology
EHR: Electronic health record
ICD-9-CM: International Classification of Diseases, Ninth Revision, Clinical Modification
ICD-10-CM: International Classification of Diseases, Tenth Revision, Clinical Modification
PDE4: Phosphodiesterase 4 inhibitor
RISE: Rheumatology Informatics System for Effectiveness
TNF: Tumor necrosis factor
tsDMARDs: Targeted synthetic DMARDs
U.S.: United States
