# Supplementary material for: Epidemiology and treatment of Behçet’s disease in the USA: insights from the Rheumatology Informatics System for Effectiveness (RISE) Registry with a comparison with other published cohorts from endemic regions
Source: Arthritis Res Ther. 2021 Aug 30;23:224. doi: 10.1186/s13075-021-02615-7 (PMC8404295; doi:10.1186/s13075-021-02615-7)
Supplement: Supplementary file 1 — Additional file 1:. Supplementary Table 1. Characteristics of patients with BD compared with all patients in the RISE registry. [file 13075_2021_2615_MOESM1_ESM.docx]

Supplementary Table 1: Characteristics of patients with BD compared with all patients in the RISE registry.

| Characteristics  mean±SD / N (%) | | Total BD patients  (N=1323) | | Total patients in RISE  (N=1,231,176) | |
| --- | --- | --- | --- | --- | --- |
| Age | | 48.7 ± 16.3 | | 61.1± 16.1 | |
| Sex | Female | 1049 | 79.3% | 909,961 | 73.9% |
| Race | White | 882 | 66.7% | 798,742 | 64.9% |
|  | Hispanic or Latino | 72 | 5.4% | 85,424 | 6.9% |
|  | Black or African American | 65 | 4.9% | 88,293 | 7.2% |
|  | Asian | 34 | 2.6% | 19,894 | 1.6% |
|  | Other^*^ | 106 | 11.9% | 238,823 | 19.4% |
|  | Unknown | 443 | 33.5% | 165,819 | 13.5% |

Abbreviations: SD: standard deviation, IQR: interquartile range, ^*^Other race includes Native Hawaiian or other Pacific Island, American Indian or Alaska Native, and multi-racial.

Total patients in RISE represents patients in RISE, aged 18 years and older, at least 2 visits with >= 30 days apart during the study period with any diagnosis.
